# Editorial Brevities

**Published:** 1887-05

**Authors:** 


### EDITORIALS AND MISCELLANEOUS.

### EDITORIAL BREVITIES.

AT the last meeting of the board of Fulton County Commissioners, Dr. Robert W. Westmoreland was elected County Physician.

DR. JAMES AVERY, who had sought a domicile in Charlotte, N. C., has returned to Atlanta. *A-very* good move; rather remove.

IT PAYS, says the World's Medical Review, to be special physician to Kaiser Wilhelm. Lauer, his medical adviser, received a donation of three hundred thousand marks on March 22.

OUR young and gifted friend Dr. F. W. McRae has just left us for New York City to avail himself of the polyclinic advantages which the great American metropolis offers to the ardent pursuer of medical science.

DEATH OF DR. HENRY WILE.—It pains us to chronicle the death of this young and skillful physician. He had been residing here about two years. His failing health prompted him to seek another clime, and to go to Colorado. He died on the way. Dr. Wile was a favorite pupil of Dr. Duhring.

THE STONE MOUNTAIN MEDICAL ASSOCIATION convened at Decatur, Ga., on the 7th of April. There was a good attendance and more than usual interest manifested on the part of members. The discussions were interesting, and a brief of them was prepared for this issue of the RECORD, but is unavoidably crowded out. It will appear in our next.

A SURPRISE—and it was an agreeable one! Mr. Parke, of the world-renowned firm of Parke, Davis & Co., stepped into our office a few days ago, in company with his genial and polite agent, Dr. Stone, now a resident of Atlanta. We were delighted to meet them. Mr. Parke is a most affable gentleman. This was his first visit to us; and he has left an impression as lasting as the monumental business which he and his associates have erected in this scientific and progressive age.

